# Bringing Down Cancer Aircraft: Searching for Essential Hypomutated Proteins in Skin Melanoma

**DOI:** 10.1371/journal.pone.0142819

**Published:** 2015-11-13

**Authors:** Mikhail Pyatnitskiy, Dmitriy Karpov, Ekaterina Poverennaya, Andrey Lisitsa, Sergei Moshkovskii

**Affiliations:** 1 Institute of Biomedical Chemistry, 119121, Pogodinskaya str, 10, Moscow, Russia; 2 ZAO Personal Biomedicine, 129164, Prospekt Mira, 124, 17, Moscow, Russia; 3 Pirogov Russian National Research Medical University, 117997, Ostrovityanova str, 1, Moscow, Russia; 4 Engelhardt Institute of Molecular Biology, 119991, Vavilova str, 32, Moscow, Russia; Universidade de São Paulo, BRAZIL

## Abstract

We propose an approach to detection of essential genes/proteins required for cancer cell survival. A gene is considered essential if a mutation with high impact upon the function of encoded protein causes death of the cancer cell. We draw an analogy between essential cancer proteins and well-known Abraham Wald’s work on estimating the plane critical areas using data on survivability of aircraft encountering enemy fire. Wald reasoned that parts with no bullet holes on the airplanes returned to the airbase from a combat flight are the most crucial ones for the airplane functioning: a hit in one of these parts downs an airplane, so it does not return back for the survey. We have envisaged that the airplane surface is a cancer genome and the bullets are somatic mutations with high impact upon protein function. Similarly we propose that genes specifically essential for tumor cell survival should carry less high-impact mutations in cancer cells compared to polymorphisms found in normal cells. We used data on mutations from the Cancer Genome Atlas and polymorphisms found in healthy humans (from 1000 Genomes Project) to predict 91 protein-coding genes essential for melanoma. These genes were selected according to several criteria, including negative selection, expression in melanocytes and decrease in the proportion of high-impact mutations in cancer compared with normal cells. The Gene Ontology analysis revealed enrichment of essential proteins related to membrane and cell periphery. We speculate that this could be a sign of immune system-driven negative selection of cancer neo-antigens. Another finding is the overrepresentation of semaphorin receptors, which can mediate distinctive signaling cascades and are involved in various aspects of tumor development. Cytokine receptors CCR5 and CXCR1 were also identified as cancer essential proteins and this is confirmed by other studies. Overall, our goal was to illustrate the idea of detecting proteins whose sequence integrity and functioning is important for cancer cell survival. Hopefully, this prediction of essential cancer proteins may point to new targets for anti-tumor therapies.

## Introduction

The recent progress in genome sequencing in the context of large cancer studies conferred a new vision on tumor as a result of mutator phenotype [1]. Instead of earlier point of view that cancer cell has mutations affecting only specific oncogenes or tumor suppressor genes it became clear that its genome is literally filled up with various somatic abberations [2]. A cancer cell clone’s survival and evolution strategy is to change its genome quickly using damage or modulation of DNA repair systems [3]. Some mutations are the drivers of the cancer process and occur in the cancer-related genes. However, most mutations occurring throughout the whole genome are not relevant to the tumor progression and represent passenger mutations. They do not help cancer cells to survive and may experience a negative selection [4].

In recent works where data from cancer genome sequencing were analyzed a pivotal attention is paid to identification of genes significantly mutated in cancer compared to the germline genome. A statistical study made on thousands of samples has reported more than 200 potential cancer driver genes [5]. The acquisition of specific mutations in these genes is the driving force of malignant transformation.

**Fig 1:**
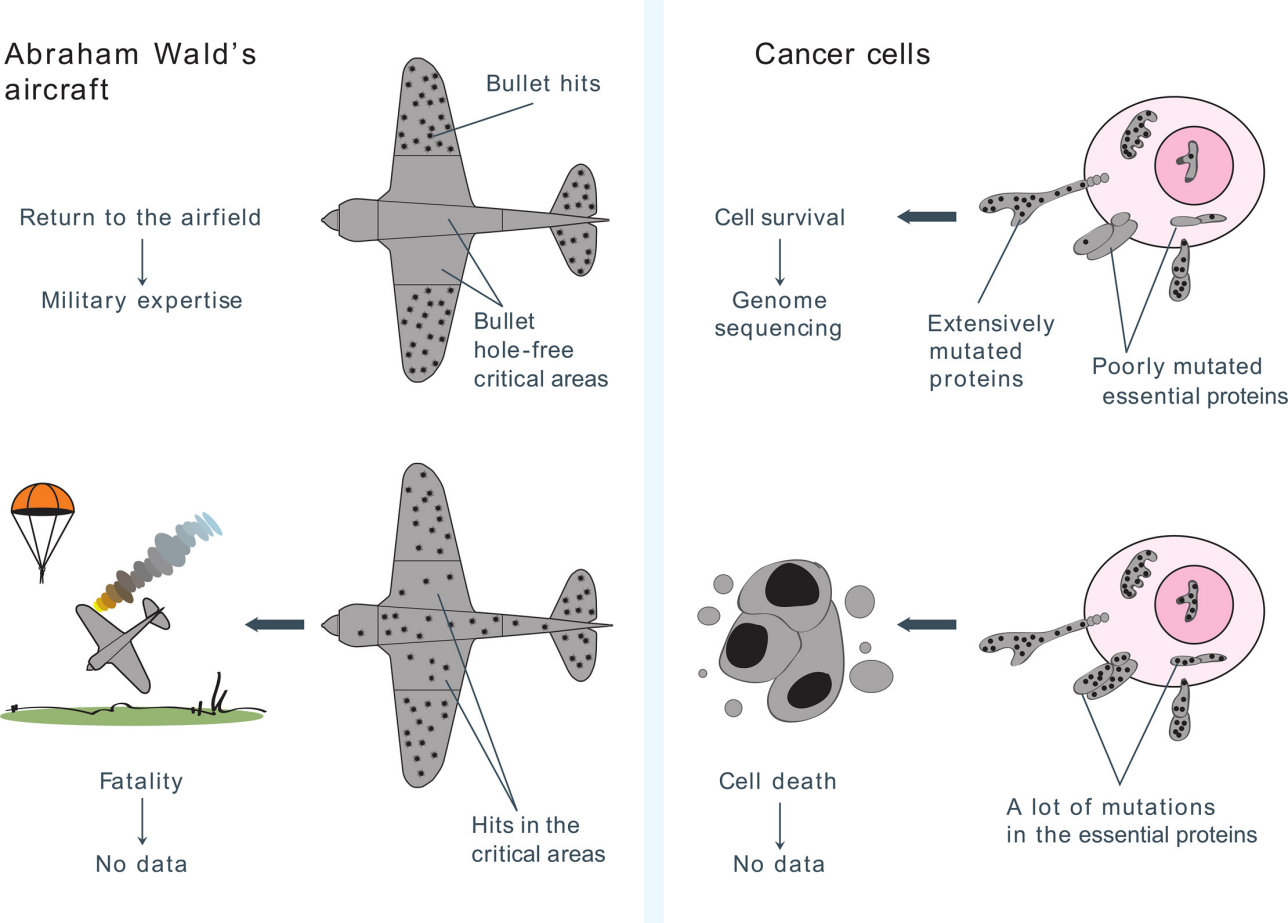
Analogy between bullet-free plane critical areas and hypomutated proteins essential for cancer. Undamaged areas on the returned planes are critical for the aircraft performance. Similarly we propose that proteins with reduced number of deleterious somatic mutations compared to germline are essential for cancer cell survival.

Our study represents an alternative approach to analysis of cancer genome data. The idea is inspired by a well-known fact from the history of applied statistics, namely Abraham Wald's aircraft problem [6]. Wald proposed to search for aircraft vulnerability zones by estimation of the bullet-free patches on airplanes which returned to the airbase from a combat flight. Indeed, it is the bullet-free areas on the machine surface are essential for the aircraft performance. If bullets hit those areas, then the machines crashed and the data on aircraft vulnerability became unobservable (Fig 1, left panel). So the bullet-free zones on the returned aircrafts were essential for the plane functioning and hence needed more armor.

We have envisaged that the airplane surface is a cancer genome and bullets are somatic mutations with high impact upon protein function. The sites with decreased number of such variants may be essential for cancer survival since the cells with mutations in these sites die and their genomes are not sequenced by cancer projects (Fig 1, right panel).

Expectedly, we were not alone in such way of thinking. In the beginning of cancer genomics, it was suggested to search homozygous DNA deletions as immutable features of cancer cells [7]. In his recent work, Polak et al. analyzed hypomutated sites in cancer genome and found them in accessible regulatory DNA due to enhanced repair in these sites [8].

However, we focused not on the cancer genome, but rather on cancer proteome. We searched for the hypomutation phenomenon from the viewpoint of protein functioning. Our goal was to identify proteins that are depleted by functionally important amino acid changes in cancer. These proteins are conserved and therefore are essential for cell survival during cancer evolution. As a reference we used polymorphisms reported in 1000 Genomes Project [9]. In fact, we have compared evolution of protein sequences during development of cancer with such evolution during 200,000 years of development of humanity from its last bottleneck [10]. We believe that our approach is able to detect proteins whose sequence integrity and functioning is more important for cancer cells than for normal tissues. Hopefully, these predicted cancer-essential proteins may represent vulnerability zones for tumor cells and hence serve as new targets for anti-cancer drugs.

## Results and Discussion

The purpose of our analysis was to identify cancer proteins under negative selection i.e. depleted with functionally important amino acid changes. These conserved proteins may be essential for cell survival during cancer evolution and hence can be regarded as possible targets for anticancer therapy (Fig 1).

For our analysis we chose the melanoma somatic mutation dataset available from TCGA website. Our approach is assumed to be more applicable to tumors triggered by point mutations rather than by copy-number alterations [11], such as lung tumors or melanomas. Those former cancers provide much more statistics on protein sequences to make the prediction robust and melanoma is characterized by the highest somatic mutation frequency compared to other cancers [12]. Also the melanoma dataset contained the highest number of variants compared to other types of cancer, total 181,175 variants.

### Defining a subset of skin melanoma proteins under negative selection

In order to find protein-coding genes experiencing negative selection during cancer evolution we used *d*_N_/*d*_S_ as an indicator of selective pressure [13]. The same logic was accepted by Ostrow et al. [14] when they studied positive selection in cancer genomes.

**Fig 2:**
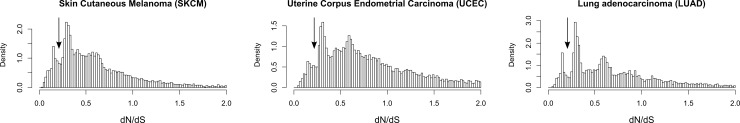
Genome-wide distribution of dN/dS ratio for three cancer types. Skin melanoma, uterine endometrial carcinoma and lung adenocarcinoma contained most of the data on cancer mutations. Local minima are reached when dN/dS is approximately 0.25 (denoted by arrows). We considered proteins with dN/dS below this threshold to experience negative selection.

We calculated dN/dS ratio for all human protein coding genes using SKCM data. Genes carrying less than 11 variants were excluded from the analysis in order to obtain more robust estimates of dN/dS ratio. Gene was considered to be under negative selection if the corresponding dN/dS value was smaller than the predefined threshold. In attempt to avoid the threshold choice by an arbitrary decision we plotted genome-wide distribution of dN/dS values for three cancer types with largest number of point mutations: two types of lung cancers and skin melanoma (Fig 2). As can be seen from the histograms, the local minima are observed at dN/dS value of about 0.25. We used this threshold as a first filter to select essential cancer genes which were supposed to be under negative selection.

Our next step was to remove genes which are not expressed in melanoma cells and hence cannot be recognized as essential for this cancer. We calculated the average expression for each gene across 374 cancer samples and removed genes with the expression levels in the lowest 20%.

Then we turned to the evaluation of genetic variants observed in SKCM and 1KG data. We divided mutations into two classes: those with high impact on the protein function and other non-significant mutations, including synonymous variations. The functional effect of non-synonymous substitutions was predicted via dbNSFP database (see Methods). Indels, stop gains/losses and splice sites variations were also classified as mutations with high functional impact.

Finally we selected genes which are depleted by functionally important variants in cancer as compared to normal tissues, i.e. the genes where fraction of mutations with high functional impact *f* for 1KG data was greater than the same fraction for cancer data, *f*_1KG_ > *f*_SKCM_. After all steps we obtained 91 protein-coding genes designated hereinafter as “essential cancer proteins”, S1 Table.

### Skin melanoma essential protein subset is significantly enriched by plasma membrane proteins: a possible link to immune surveillance

By manual looking through the list, one could mark some distinct categories among those proteins (Table 1). Most represented categories include membrane transport proteins, such as ion channels and solute carriers, neural proteins of various functions, cell adhesion molecules, etc. In order to describe the resulting list more formally we performed functional enrichment analysis using WebGestalt website [15].

**Table 1:** List of protein-coding genes with amino acid sequences under negative selection in skin melanoma genomes (essential cancer proteins). Genes were filtered as described here. Categories were defined by manual biocuration.

Category	Gene names
Membrane transport	ABCA3, CATSPER1, SLC12A8, SLC24A1, SLC26A7, SLC27A5, SLC45A1, SLC5A6, SLC9A3, TMC7
Neuronal contacts, synapses	CADPS2, EPB41L1, MRGPRX3, MRGPRX4, NRP2, PLXNA2, SEMA4F, SEMA6C, THBS4, UNC5B
Cell contacts, adhesion	CDH11, GJB4, GJB5, ITGB5, LRFN1, LRG1, PCDHB13, PCDHGA12, PCDHGC5
Metabolic enzymes	AKR1B1, ENGASE, GCNT3, INPP5B, NMNAT3, NNT, TGM5, UBIAD1
Proteases and peptidases	ADAMTS15, BMP1, CAPN11, CAPN12, CASP10, PM20D1, TMPRSS9
Nucleic acid binding	ADAR, ERCC6, NFATC2, ZNF195, ZNF493, ZNFX1
Receptor for cytokines and hormones	CCR5, CXCR1, EDAR, MC2R, TNFRSF10A
Protein kinases	DAPK2, MLKL, PTK2B
Cancer pathway related	BCL2L12, MYCT1, RSPO1
Other	CRISPLD2, CYP2J2, EPB41L4B, FGF5, GPR115, HBB, IPO13, LRRC15, MIB2, MYH9, MYO18A, NVL, OR2C3, PDIA4, PDPR, PLD2, PVRL4, RHPN2, SH3BP4, SMYD1, STEAP3, STK11IP, STOX1, TBC1D9B, TMEM104, TMEM63B, TTC7A, UNC45A, VPS18, XPO6

**Fig 3:**
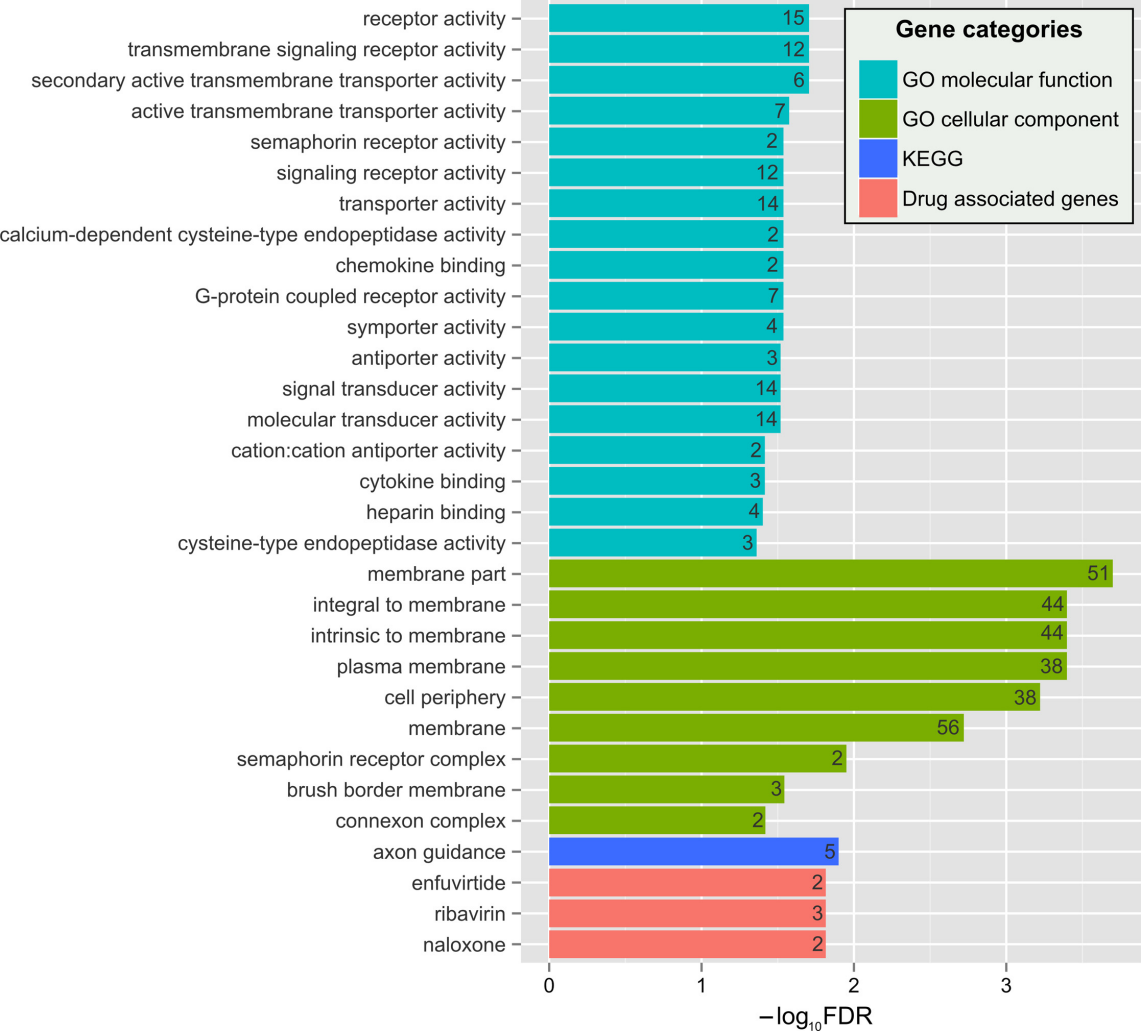
Enrichment of the essential cancer protein subset by Gene Ontology, KEGG and PharmGKB drug target categories. P-values were adjusted using Benjamini-Hochberg correction for multiple comparisons. Number of essential proteins falling into category is depicted within each bar.

**Fig 4:**
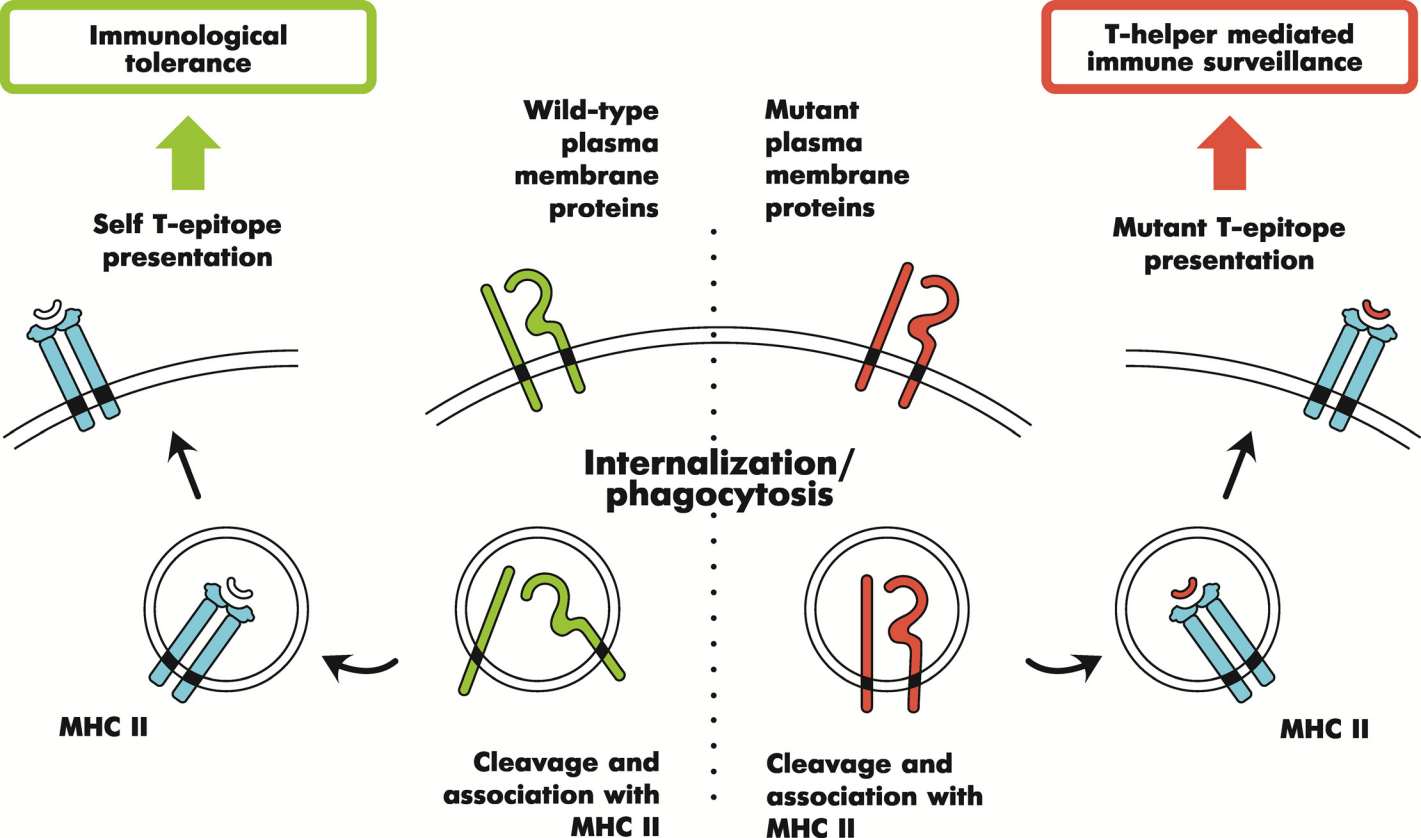
Hypothetical scheme for negative selection against neoantigens derived from cell surface proteins. Preferential involvement of surface proteins to MHC-restricted antigen presentation is known in the art [20]. Cancer cells exposing MHC-II epitopes with mutated antigens are more likely to be eliminated by T-cell mediated immune surveillance.

The enrichment of 91 essential protein subset by Gene Ontology [16], KEGG [17] and PharmGKB drug target [18] categories had shown a significant trend towards membrane proteins, specifically, proteins of plasma membrane and cell periphery (Fig 3, S2 Table). Obviously, such general categories cannot fully decipher possible molecular pathways or cell proliferation mechanisms. However, there may be a mechanistic interpretation of the overrepresented plasma membrane proteins. It is widely recognized that cancers escape from host immunity through evolution of cancer clones [19]. We have hypothesized that one of the mechanisms leading to conservation of plasma membrane and cell periphery protein sequence in melanoma could be a result of such immune escape. More specifically, high impact mutations in cell periphery and plasma membrane proteins may lead to formation of major histocompatibility complex (MHC) II-dependent neo-antigens [20]. MHC II-restricted protein epitopes reactive to T-helper lymphocyte subpopulation, as widely known, are formed by digestion of phagocytized extracellular proteins and cell periphery proteins accompanying internalized membrane parts. The fact that such mutations are depleted in cell surface and periphery proteins reflects escape of melanoma cells from CD4^+^-T-cell mediated immunity. Recently it has been reported that adoptive immunity induced against a T-helper-1 (MHC II-restricted) neo-antigen epitope provided tumor regression in a patient with metastatic cholangiocarcinoma [21]. Significant response of CD4+-T-cells against personalized tumor neo-epitopes was also found in patients with metastatic melanoma [22]. Thus, we may observe the enrichment by cell periphery proteins in target subset as a result of purifying selection against formation of neo-antigen T-helper epitopes. When a plasma membrane protein is extensively mutated in a cancer cell, it is digested after vesicle internalization and its mutant peptides bind MHC II as neo-epitopes which are not recognized by immune system as self-epitopes. Having such neo-epitopes expressed, a cancer cell cannot avoid the immune surveillance and is eliminated (Fig 4). Notably, despite MHC II itself is expressed in limited cell types, such as professional antigen-presenting cells, melanoma cells are reported to express various types of the receptor [23].

Immunoediting of the cancer genome against antigen formation as described recently [24] is in a good correspondence with our hypothesis.

### CCR5 and CXCR1 chemokine receptors as essential melanoma proteins

One of the proteins in our subset is a C-C chemokine receptor type 5 (*CCR5*), the chemokine receptor which was extensively studied due to its ability to provide HIV fusion with the target cell. Moreover, a deletion of this protein known as CCR5-Δ32 protects its carrier against selected strains of the virus [25]. Thus, in case of changed amino acid sequence of the receptor it does not serve as a cofactor for the virus particle. Drugs blocking CCR5, e.g. maraviroc, are considered as a therapy of choice for the treatment of HIV infection.

At the same time, a role of CCR5 in cancer has been widely discussed. It is known that this receptor is often overexpressed in some cancer types [26]. CCR5 expression has been induced after oncogenic transformation of cells [27]. Evidences are accumulating that this receptor’s signaling through its ligand, CCL5, provides cancer progression, e.g. metastasis. CCR5 antagonist drugs have been shown to block metastasis of basal breast cancer [28] and Src-induced prostate cancer [29] in mice. It was proposed to use well-tolerable CCR5 inhibitor drugs, which are already approved for use in HIV infection, in clinical trials for metastatic cancers [28]. An observational trial that studied the effect of maraviroc on metastatic colorectal cancer had been completed, but no results have been reported yet [29].

As for melanoma, it was found that functional CCR5 is important for progression of this tumor: CCR5^−/−^ mice show reduced tumor volume and increased survival rate compared to wildtype mice [30, 31]. Vivanco et al. also reported that melanoma growth and metastasis was inhibited in CCR5^−/−^ mutant mice [32]. The authors have proposed that CCR5/CCL5 signaling negatively affects CD8 T-lymphocyte effects. Thus, melanoma cells expressing functional CCR5 may thereby contribute to the immune escape.

The fact that C-C chemokine receptor type 5 is one of essential cancer proteins positively illustrates applicability of our approach. This receptor is a druggable cancer target, at least, for adjuvant therapy.

Another important cytokine receptor in our list is CXCR1. It binds interleukin-8 protein (CXCL8) which is a major chemotaxis agent in innate immunity. Notably, CXCR1 and above-mentioned CCR5 use the same signaling pathways and are closely related in their function [33]. As for the latter, CXCR1 is known to be used by melanoma cells for outgrowth and metastasis [34]. Recently, it was shown that elevated expression of this receptor is correlated with tumor malignancy [35]. Most likely, CCR5 and CXCR1 are used by melanoma cells to survive in inflammatory environment.

### Semaphorin pathway is potentially important for melanoma survival

Another promising enrichment among the essential cancer proteins is related to the semaphorin receptor complex and axon guidance pathway (Fig 3, S2 Table). Semaphorins are involved in cell guidance during development and in adult tissues [36]. These proteins were recently shown to function as suppressing and promoting agents in various cancer types via transactivation of receptor tyrosine kinases [37]. Notably, both types of semaphorin-binding receptors, plexins and neuropilins, are represented in our list and may be involved in cancer cell survival [38]. Plexin A2 (*PLXNA2*) protected cancer cells from death after human papillomavirus infection [39]. For neuropilin-2 (*NRP2*), there is an evidence of its role in malignant melanoma where its expression was correlated with tumor progression [40]. These results provide a background for further experimentation to discover cancer drug targets among semaphorin complex proteins [36].

### Protein interactions between essential melanoma proteins

In order to understand how predicted essential melanoma proteins cooperate with each other, we analyzed corresponding protein-protein interactions using STRING database [41]. We have selected only high-confident interactions with score greater than 0.9. Interacting preys were reported for 46 of 91 genes of interest (Table 2). Most of protein interactions are known for cytokine receptors CCR5 (115 preys) and CXCR1 (77 preys), of them about 70 interactions involve common partners. Another pair of interactors which are characterized by many common partners consists of caspase-10 (CASP10) and TRAIL-R1 death receptor (TNFRSF10A).

**Table 2:** Protein-protein interactions: the number of preys reported for essential melanoma proteins as baits according to STRING database. Only highly reliable interactions with score greater than 0.9 were considered.

Gene name(s)	Number of interacting preys
CCR5	115
CXCR1	77
PLD2	45
NFATC2	44
INPP5B	36
PTK2B	35
CYP2J2	31
MC2R	27
ERCC6	26
ADAR	25
ITGB5	24
AKR1B1, CASP10, PLXNA2	15
CDH11, OR2C3	12
GCNT3, NMNAT3	11
TNFRSF10A	10
UNC5B	8
BMP1, HBB, MYH9	7
NRP2, SLC27A5	6
EPB41L1, FGF5, IPO13, SLC9A3	4
PVRL4, RSPO1, STEAP3, THBS4, VPS18	3
ABCA3, CATSPER1, NNT, PDIA4, STK11IP, UBIAD1	2
EDAR, MIB2, MLKL, MYCT1, MYO18A, UNC45A	1

**Fig 5:**
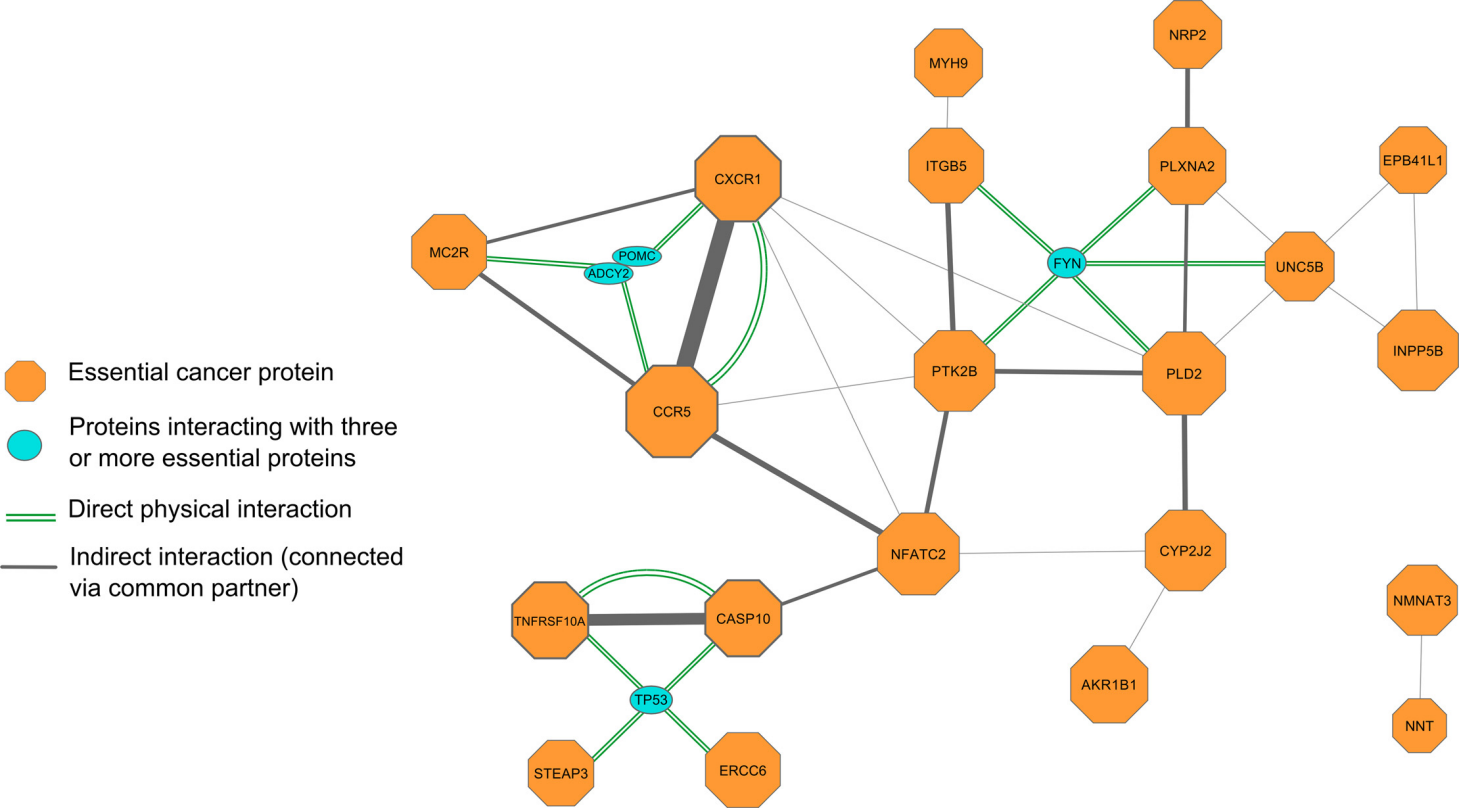
Interaction map for essential melanoma proteins. The map was built using STRING database with high-confidence interaction score threshold 0.9. Size of the octagon is proportional to the number of partners of corresponding protein. Line width reflects the number of common partners between two baits. Doubled green lines denote known direct physical protein-protein interactions. Prey proteins interacting with three or more baits are shown as blue ovals: FYN, TP53, ADCY2 and POMC.

The interaction map of proteins hypomutated in melanoma (Fig 5) can be easily subdivided to several main networks. First, three G-coupled receptors CCR5, CXCR1 and MCR2 form a triangle of many interactions. A role of these receptors in melanoma where they probably promote cancer cell survival and metastasis is partly described above.

The largest network component between integrin ITGB5, protein kinase PTK2B, semaphorin receptors (PLXNA2) and phospholipase D2 (PLD2) is defined by their interactions with FYN protein. The latter is a well-known protein kinase from the Src family and is involved in the series of cellular functions, such as T-cell immunity, axon guidance and is also considered as a potential proto-oncogene [42]. Little is known about its function in melanoma and there is some evidence that Fyn cannot be a medication target in advanced melanoma. In particular, when it was inhibited by saracatinib drug along with other Src kinases, no benefit was observed in advanced melanoma [43]. However, based on our data, more attention should be paid to study of Fyn role in melanoma separately from other Src kinases, such as Src itself, Yes, etc.

Unexpectedly, amongst hypomutated proteins, some partners of TP53 tumor suppressor were found. They include, inter alia, physically interacting proteins, caspase-10 (CASP10) and TRAIL-R1 death receptor (TNFRSF10A). In contrast to these results, components of TRAIL apoptosis pathway are considered to have antitumor effect, when active [44]. This contradiction awaits further deciphering.

## Conclusions

The cancer genome concept developed over the past decade has literally revolutionized our understanding of cancer molecular biology [45]. Results of cancer genome projects are especially promising for evidence-based and personalized medicine, disclosing driver genes which provide tumor development and progression [5]. At the same time, not all driver proteins may serve as targets for drug therapies. Many cancers are primarily caused by tumor suppressors which are irreversibly inactivated by gene mutations. In these cases, drug therapy represents a big challenge due to difficulties in complementation of the lost function [46]. Therefore, search for new drug targets is the primary task of post-cancer-genome studies.

Positive selection regulates, at the level of corresponding genes, amino acid sequences of driver cancer proteins during clone competition accompanying tumorigenesis. Necessity of oncogenes and tumor suppressors to be mutated in most cancers inspires researchers to implement scoring systems to select hypermutated driver genes [5]. In contrast to these useful efforts, instead we focused on proteins whose corresponding genes are hypomutated in tumor, i.e. experience negative selection during cancer evolution. We believe that these proteins may provide additional drug targets especially in the cases where cancer drivers represent suppressors functionally destroyed by mutations.

With our approach aggregating evolutional dN/dS parameter as a measure of negative selection, gene expression and functional impact of amino acid changes, we have predicted 91 protein-coding genes to be essential in melanoma and found several significant enrichments. For example, the list contained increased number of cell surface and cell periphery proteins. In our opinion, it could be a sign of immune system-driven negative selection of cancer neo-antigens [22]. Furthermore, some examples of hypomutated proteins represent known cancer-related proteins, such as cytokine receptors CCR5 and CXCR1 [33].

It should be emphasized that the results of our research strongly depend on the available sample size. The more mutation data is accumulated for protein, the more confidently we can designate whether it is essential or not. Essential genes are characterized by low frequency of high-impact mutations [47] which also may decrease the power of our approach. Hence for the analysis we have chosen skin melanoma exome dataset [48], because this cancer is characterized by the highest level of point mutations. We understand that currently available amount of data may be insufficient to identify all the essential proteins for this type of cancer.

Although our data is preliminary, this work is mostly intended to illustrate a general idea of defining essential cancer-specific proteome. Results may become more reliable when the larger number of individual cancer genomes will be accumulated. However, even in its present form our list of predicted essential melanoma proteins provides a background for targeted experimentation with tumor cell survival by blocking the protein candidates *in vitro* and *in vivo*.

## Methods

Cancer mutation data were downloaded from the Synapse website [49], accession number syn1729383. These data initially were obtained via The Cancer Genome Atlas [50] and were reprocessed to filter out false positives and germline variants, details can be found in [51]. Skin cutaneous melanoma dataset (SKCM) had the highest number of variants compared to other datasets, total 181175 variants. Data on germline polymorphisms were downloaded from the 1000 Genomes (1KG) website [9] and annotated using ANNOVAR software [52]. Final table contained 425069 variants located within the coding regions. Genes with less than 11 variants either in SKCM or in 1KG data were removed from subsequent analysis.

For each human protein-coding gene the ratio of the rates of non-synonymous and synonymous substitutions (dN/dS) was calculated as in [14] using SKCM data on somatic mutations. Genes with dN/dS value greater than 0.25 (i.e. experiencing either neutral or positive selection) were removed.

Gene expression data were obtained via the TCGA website and contained information about 20531 genes expressed in 374 melanoma samples. The gene expression profiles that had absolute expression levels in the lowest 20% of the dataset were removed.

Functional impact of non-synonymous single nucleotide variants in both SKCM and 1KG data was assessed using dbNSFP resource [53]. This database compiles results from ten prediction algorithms (including SIFT, Polyphen2 and MutationAssessor) into two ensemble scores, MetaSVM and MetaLR. Larger value of score indicates that the variant is more likely to be damaging. We considered variant to have high impact on protein functioning if either MetaSVM or MetaLR value was greater than 70-th percentile of corresponding score. Somatic mutation was also considered to have high functional impact if it belonged to one of the following classes: insertion/deletion, stop loss, stop gain, or splice site mutation. For each gene we calculated fraction of mutations with high functional impact *f*_1KG_ and *f*_SKCM_ as *f* = *#(high func*. *mutations) / #(total mutations)*

Functional geneset enrichment analysis via hypergeometric test was performed using WebGestalt software [15]. As a reference set we supplied list of 16172 genes with average SKCM expression greater than 20-th percentile. Correction for multiple testing was done using Benjamini & Hochberg method.

A list of interactions for selected proteins was downloaded from the STRING database v. 9.1 [41] using the Python script. STRING collects various associations between proteins, such as structural predictions, textmining, pathway analysis and experimental results from other web resources, to form its aggregative score. High-confident interactions with the score greater than 0.9 were used for analysis. Network visualization and analysis was performed via Cytoscape [54].

## Supporting Information

S1 Table List of protein-coding genes with amino acid sequences under negative selection in skin melanoma genomes (“essential” cancer proteins). Column description: Gene, EntrezGene: gene identifiers according to HGNC and NCBI Gene. Description: gene function description. skcm.dnds: dN/dS ratio calculated using SKCM data on somatic mutations. quantile.expression: percentile of gene expression (TCGA SKCM data). norm.neutral: number of synonymous SNVs (1KG data). norm.high: number of mutations with high impact functional impact (1KG data). skcm.neutral: number of synonymous SNVs (TCGA SKCM data). skcm.high: number of mutations with high impact functional impact (TCGA SKCM data).
(TSV)

S2 Table Enrichment of the essential cancer protein subset by Gene Ontology, KEGG and PharmGKB drug target categories. P-values were adjusted using Benjamini-Hochberg correction for multiple comparisons.
(TSV)
